# Insights from the protein-protein interaction network analysis of Mycobacterium tuberculosis toxin-antitoxin systems

**DOI:** 10.6026/97320630013380

**Published:** 2017-11-30

**Authors:** Zoozeal Thakur, Renu Dharra, Vandana Saini, Ajit Kumar, Promod K. Mehta

**Affiliations:** 1 Centre for Biotechnology, Maharshi Dayanand University (MDU), Rohtak-124001 (Haryana), India;; 2 Toxicology & Computational Biology Group, Centre for Bioinformatics, Maharshi Dayanand University (MDU), Rohtak-124001 (Haryana), India;

**Keywords:** Mycobacterium tuberculosis, Toxin-antitoxin, STRING, Cytoscape, Homology Modeling

## Abstract

Protein-protein interaction (PPI) network analysis is a powerful strategy to understand M. tuberculosis (Mtb) system level physiology in the identification of hub proteins. In the present study, the PPI network of 79 Mtb toxin-antitoxin (TA) systems comprising of 167 nodes and 234 edges was investigated. The topological properties of PPI network were examined by 'Network analyzer' a cytoscape plugin app and STRING database. The key enriched biological processes and the molecular functions of Mtb TA systems were analyzed by STRING. Manual curation of the PPI data identified four proteins (i.e. Rv2762c, VapB14, VapB42 and VapC42) to possess the highest number of interacting partners. The top 15% hub proteins were identified in the PPI network by employing two statistical measures, i.e. betweenness and radiality by employing cytohubba. Insights gained from the molecular protein models of VapC9 and VapC10 are also documented.

## Background

The ability of Mycobacterium tuberculosis (Mtb) to persist inside the host cells under a variety of adverse conditions including oxidative stress, nutrient starvation and hypoxia helps to understand pathogenesis [1, 2]. Mtb toxin-antitoxin (TA) systems comprising of two component genetic modules - a stable toxin and relatively unstable antitoxin, play a significant role for the survival of bacteria under stress conditions. Mtb harbors a high number of TA systems (79) belonging to various families such as VapBC, MazEF, ParDE, higBA, RelBE, and several uncharacterized TA systems [3]. These TA systems are associated with antibiotic resistance, biofilm formation and persistence inside the host cells [4]. In response to stress conditions, the labile antitoxin is degraded and toxin is released, which in turn halts transcription, translation etc. and that leads to growth inhibition and even cell death [3].

Protein-protein interaction (PPI) is imperative to many cellular process including signal transduction, transcriptional regulation, post-translational modification, etc. [5]. PPI network analysis is a robust approach to understand the mechanisms associated with mycobacterial pathogenesis, functional annotation of genes, etc. [6, 7] PPIs can be detected by computational and experimental methods. Experimental methods include yeast two-hybrid system, tandem affinity purification and protein microarrays, whereas computational methods include interlog-based method and prediction based on genetic algorithms. In comparison to experimental techniques, computational methods take less time and are inexpensive [8]. In the present study, we report the topological and functional enrichment analysis of PPI network of 79 Mtb TA systems constructed from STRING v10.5 and Cytoscape v3.5.0. The molecular models of VapC9 and VapC10 have also been documented to gain functional insights.

## Methodology

### Literature mining of Mtb TA system genes

**Figure 1:**
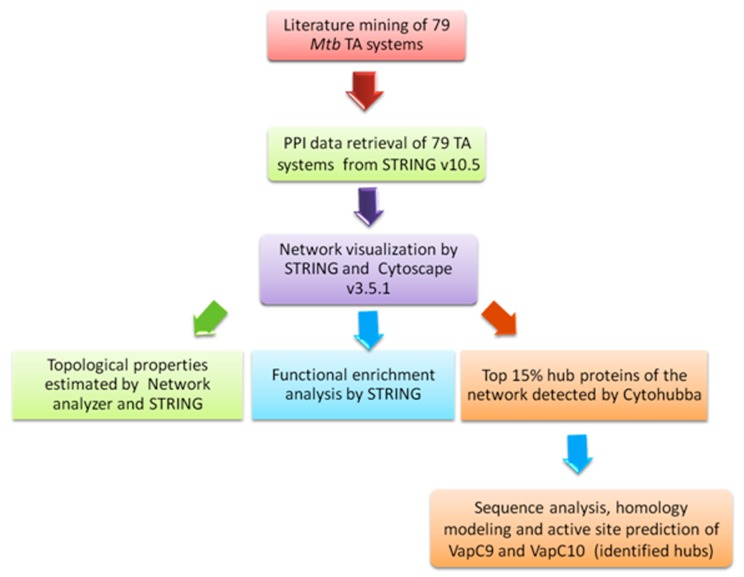
A flowchart representing the methodology applied in the study; arrows represent flow of information and transition from one step to another

A total of 79 Mtb H37Rv TA system genes enlisted in Suppl Table 1 were mined from the literature [3]. The mined 79 TA systems belonged to various TA families including VapBC (50 members), MazEF (10 members), HigBA (3), ParDE (2), RelBE (2), YefM/YoeB (1) and 11 unclassified TA system genes Figure 1.

### PPI Network construction

The candidate 79 Mtb TA systems were converted into seed sequences to mine PPI data from STRING (Search tool for the Retrieval of Interacting Genes/Proteins) v10.5 database (http://string-db.org) [9]. Interaction sources selected for generation of PPI network were text mining, experiments, databases, co-expression, neighborhood, gene fusion, and cooccurrence. PPIs that possessed at least a medium confidence score of 0.400 were considered for network generation. Network construction and visualization was done by cytoscape v 3.5.1 [10] and STRING v10.5.

### Topological and functional enrichment analysis of PPI network

The topological parameters of PPI network, i.e. number of nodes, number of edges, average node degree, etc were evaluated by STRING v10.5. The protein-protein association data obtained from STRING was further utilized to compute several other topological parameters such as average clustering coefficients, topological coefficients, and shortest path lengths etc. via Network analyzer, a cytoscape plugin app, by treating the network as directed graph. In addition, functional enrichment of input seed sequences of Mtb TA systems was carried out by STRING to identify significantly enriched GO (Gene Ontology) biological processes and molecular functions.

### Identification of Hub proteins

Cyto-Hubba [11], a java plugin for Cytoscape software, was employed to determine the hub proteins of PPI network of Mtb TA systems. In this study, two centrality measurements, i.e. betweenness and radiality were applied to mine the top 15% hub proteins of the network. In addition, we further mined hub proteins, which were commonly identified by both the algorithms.

### Sequence analysis of VapC9 and VapC10

Protein sequence information of VapC9 and VapC10 was retrieved from Tuberculist database [12]. Various domain identification tools including InterProScan [13], Pfam [14], and NCBI CDD (Conserved domain database), [15] were employed to detect the conserved domains present in the protein sequences, which in turn carried out sequence similarity search with the close orthologus family members. The physical and chemical properties such as extinction coefficient, instability index, and aliphatic index, GRAVY etc. were determined by Protaparam tool of ExPASy [15, 16].

### Homology modeling of VapC9 and VapC10

BLASTp search with default parameters was carried out against PDB database to identify the suitable templates for construction of homology models of VapC9 and VapC10. The search identified 2FE1 (resolution 2.2 Å) and 2H1C (resolution 1.8 Å) as the best suitable template structures for VapC9 and VapC10, respectively. Modeler v9.17 was utilized for the construction of homology model of VapC9 and VapC10. The energy minimization of constructed 3D models was performed by chimera v1.11.2.

### Model evaluation

Energy minimized 3D models of VapC9 and VapC10 were subjected to various model validation servers to evaluate the stereo chemical properties. To determine parameters such as Zscore, QMean Score, D-fire Energy and residue by residue geometry, energy minimized theoretical models were subjected to SWISS-MODEL server [17]. Various other model evaluation tools were also employed, i.e. ERRAT [18], ProQ [19], Molprobity [20], RESPROX [21] and ProSA-web [22] to determine the model quality.

### Active site prediction

Metapocket 2.0 was employed to determine active site of VapC9 and VapC10 [23]. Metapocket uses consensus approach to detect ligand-binding sites by employing eight methods: LIGSITE, PASS, SURFNET, Fpocket, GHECOM, ConCavity, POC ASA and Q-Sitefinder.

## Result and Discussion

### PPI network analysis of Mtb TA systems:

**Figure 2:**
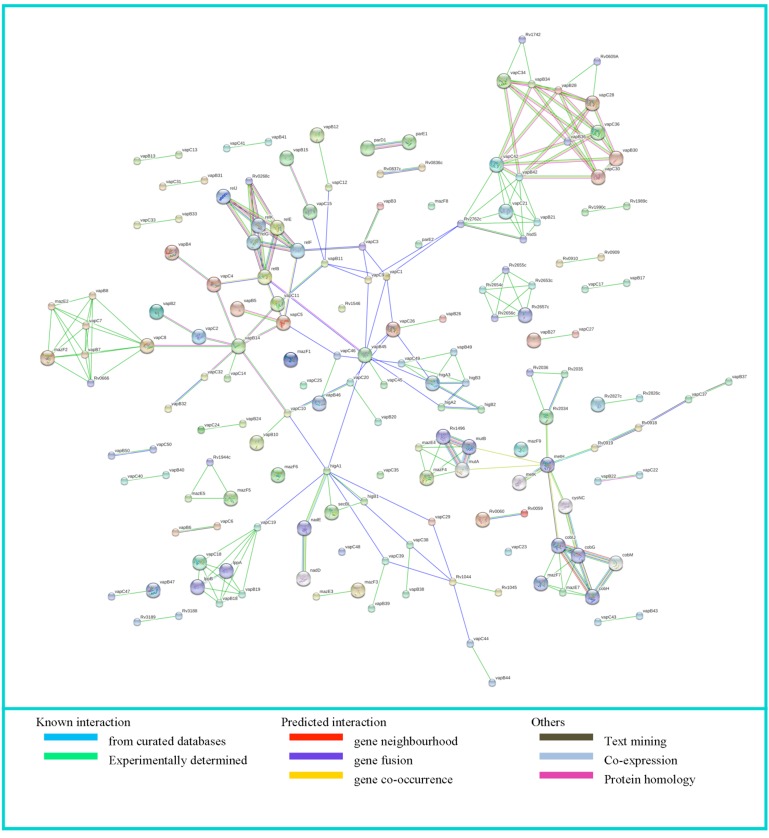
Protein-protein interaction network obtained and visualized by STRING v10.5 for input 79 Mtb TA systems. Nodes depict proteins and PPI are represented by edges in the network; interaction source of the PPI's are represented by various colors.

Protein-protein association data of 79 TA systems of Mtb H37Rv was extracted from STRING v 10.5. To completely explore the PPI data, the search was set to include all the source parameters. The mined PPI data was comprised of total 468 PPI's as depicted in Figure 2. Manual curation of the PPI data revealed that 63 proteins out of 468 PPIs possessed ≥4 interacting partners in the network (Suppl Table 2), whereas 64 proteins were associated with only one interacting partner. Two groups of proteins, comprising of 20 and 15 proteins were highly connected with each member having five and six interacting partners, respectively. Notably, two members of VapBC family, i.e. antitoxin VapB45 and antitoxin VapB14 were found to possess 8 interacting partners each. Interestingly, both the toxin and antitoxin of VapBC42 were highly connected with each possessing 9 interacting partners. Strikingly, antidote HigA1 possessed the highest number (10) of interacting partners. For 17 proteins, no PPI information could be extracted from STRING. PPI information extracted from STRING v10.5 ranged from medium to highest confidence scores. In fact, 84 (~18%) of the protein-protein associations fell within highest confidence interval (CI, S > 0.9), 176 (37.6%) within high CI (0.7 ≤ S < 0.9), and 208 (44.44%) within medium CI (0.4 ≤ S < 0.7).

In addition, topological and functional enrichment analysis of input Mtb TA systems was carried out by STRING and 'network analyzer' a cytoscape plugin. Network statistics obtained by STRING database revealed that the extracted interactome was comprised of 167 nodes and 234 edges. The average node degree and average local clustering coefficient of the network was determined to be 2.8 and 0.628, respectively (Table 1). On the other hand, 'network analyzer' a cytoscape plugin estimated several other topological parameters such as network diameter, network radius, shortest path, characteristic path length and average number of neighbors (Table 1). GO biological process and molecular function enrichment analysis of input seed sequences was carried out by STRING v10.5. The majority of Mtb TA system proteins were significantly enriched in biological processes associated with regulation of growth (1.46E-77), nucleic acid phosphodiester bond hydrolysis (1.08E-61), RNA phosphodiester bond hydrolysis (3.73E-60) and negative regulation of growth (2.65e-45, Table 2). Furthermore, molecular functions of such proteins were primarily related with nuclease activity (6.27e-62), ribonuclease activity (7.72e-61), and metal ion binding (7.36e-12, Table 3). Similar to gene enrichment analysis of this study, activation of TA systems leading to growth arrest by the toxin partners of VapBC, RelBE, MazEF, and HigBA families has also been reported [3].

Hub proteins of PPI network represent highly connected nodes with special biological properties and are more evolutionary conserved than non-hubs. In fact, removal of such hubs can lead to network disruption and thus are considered as attractive drug targets [24, 25]. Identification of hub proteins can be carried out by in silico tools such as Hubba, cytohubba, and CHAT etc. [26, 27]. In the present study, cytohubba was used to explore the hub proteins of Mtb TA systems PPI network. The top 15% hub proteins were identified on the basis of radiality and betweenness algorithms (Table 3, 4). HigA1 and VapB45 antitoxins were determined to be the top scorer hub proteins by betweenness and radiality method, respectively. Antitoxin HigA1 (Rv1956) belonging to HigBA family has been reported among the 10 top most upregulated Mtb TA systems of drug tolerant persisters [3]. On the other hand, VapB45 (Rv2018) is the antitoxin partner of VapC45, but no experimental data is available to elucidate their role in Mtb pathogenesis. In addition, we mined the hub proteins that were commonly identified by both the algorithms (Table 4). Notably, majority of the hub proteins identified belonged to the VapBC family apart from the members of HigBA and RelBE families. VapBC is the largest family of Mtb TA systems characterized by the presence of PIN domain and functions mostly as ribonucleases [3, 28]. Interestingly, Rv2762, which was not part of input seed proteins, was also detected as a top ranker hub protein.

**Figure 3:**
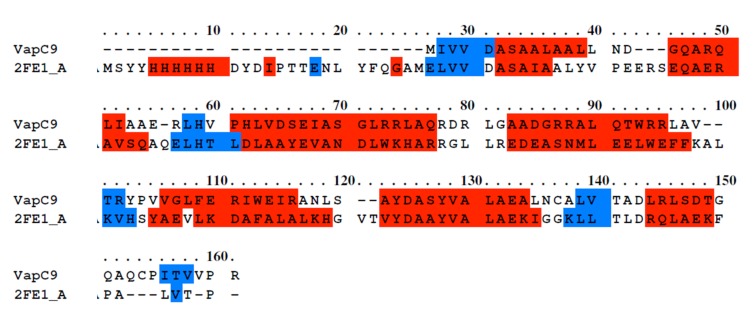
Sequence alignment of VapC9 with 2FE1_A obtained by PRALINE. Red color represents helix and blue color represents strand predicted by DSSP and PSIPRED.

**Figure 4:**
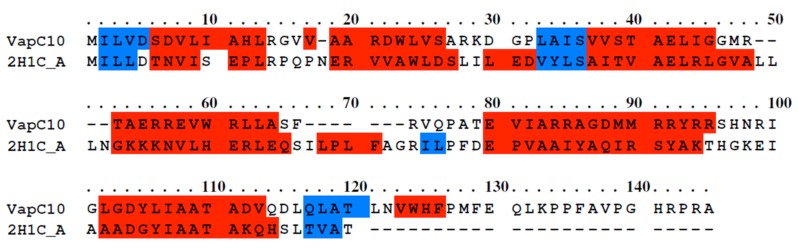
Sequence alignment of VapC10 with 2H1C_A obtained by PRALINE. Red color represents helix and blue color represents strand predicted by DSSP and PSIPRED.

Antitoxins are reported to be small proteins with less order in their structure. Therefore, it is difficult to find druggable pockets on their surface that can accommodate small-molecule inhibitors, whereas toxins are more stable and ordered in their structure, and are also considered as attractive targets for drug-design [28]. Therefore, we focused to elucidate the structural insight of toxins identified by in silico analysis. It was found that 5 out of 10 top ranking hub proteins were antitoxins, i.e. higA1, VapB45, VapB14, VapB11 and RelB, whereas three proteins were toxins, i.e. VapC1, VapC9 and VapC10. Since the structure of VapBC1 is available at PDB, we focused to determine the structural insights of VapC9 and VapC10 proteins. Domain identification tools used in the study, i.e NCBI CDD, Pfam and InterProscan revealed the presence of PIN domain in both VapC9 and VapC10. In fact, PIN domains are small protein domains of ~130 amino acids, which are characterized by the presence of three invariant amino acid residues and fourth lesser-conserved acidic residue [3, 29]. Physicochemical properties were computed by protparam tool of ExPASy for VapC9 and VapC10 (Table 5). Protein BLAST was carried out against PDB database to identify homologs with resolved 3D structure for structure prediction of VapC9 and VapC10. The crystal structure of PAE0151 from Pyrobaculum aerophilum (2FE1) was selected as the best suitable template on the basis of maximum query coverage (100%) and maximum identity (79%) for input VapC9 protein sequence (Figure 3). In a similar manner, crystal Structure of Fitacb from Neisseria gonorrhoeae (2H1C) was chosen as the best template structure for the development of homology model of VapC10 based on maximum sequence coverage (81%) and maximum sequence identity (32%) (Figure 4). Five theoretical models generated for each protein by Modeler v9.17 were assessed on the basis of DOPE score and GA341 score. The model with highest GA341 score and lowest DOPE score was selected and was further subjected to energy minimization by Chimera v1.11.2.

**Figure 5:**
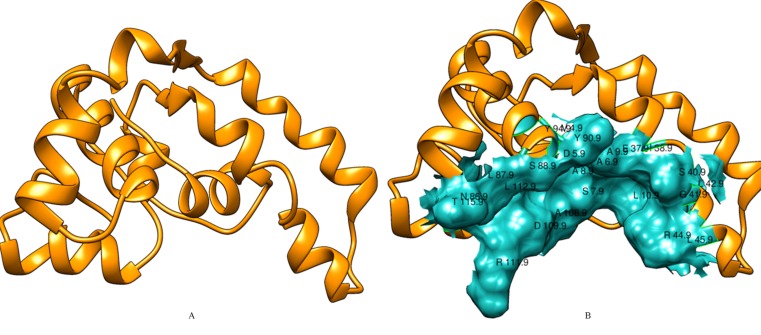
(A) 3D model of VapC9 built by Modeler 9.17 and energy optimized by Chimera v1.11.2. (B) Active site of VapC9 determined by Metapocket v2.0; active site residues are labeled with amino acid identifier and residue number.

**Figure 6:**
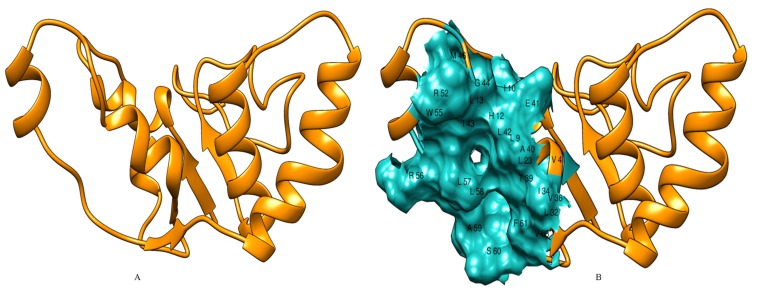
(A) 3D model of VapC10 constructed by Modeller 9.17 and energy optimized by Chimera v1.11.2. (B) Active site of VapC10 determined by Metapocket v2.0; active site residues are labeled with amino acid identifier and residue number.

Theoretical models of VapC9 and VapC10 were subjected to various structural validation servers to assess the correctness of the models. Ramachandran plot obtained for VapC9 by PROCHECK of swiss model server revealed that 93 % of amino acid residues were present in most favored regions, 6.1% in additionally allowed regions and 0. 9 % in generously allowed regions (Table 6). On the other hand, Ramachandran plot for VapC10 showed that 84.5 % residues were present in the most favored region, 12.9 % in additionally allowed region and 2.6 % in generously allowed region (Table 6). Notably, for both the generated models, none of the amino acid residues was observed in the disallowed region. Overall G-factor score computed for VapC9 and VapC10 by swiss model server fell in the acceptable cut-off range. In addition, Z-score, Q-mean score and D-fire score were also estimated by swiss model server, which further confirmed the reliability of the models generated (Table 6). The overall quality factor score obtained after ERRAT analysis was 97.3 % for VapC9 and 93.5 % for VapC10. Prosa web Z score for VapC9 and VapC10 was -4.07 and -3.64 respectively, thereby suggesting that the structures are of good quality (Table 6). Predicted resolution by resprox along with LG and Maxsub score determined by ProQ also indicated the reliability of 3D models. In addition, MOLPROBITY revealed that none of the residue possessed bad bonds or β deviations > 0.25A (Table 6). The 3D models generated in the study were of reliable quality as assessed by various structural assessment reports such as PROCHECK of swiss model server, ERRAT, ProSA-web, ProQ, MOLPROBITY and ResProx. In addition, active site of VapC9 and VapC10 was identified by Metapocket v2.0. The generated homology models and active site determined by metapocket v2.0 of VapC9 and VapC10, respectively are shown (Figure 5A, B, Figure 6A, B). The developed models can be used for structure based drug designing.

## Conclusion

We reported the construction and extensive analysis of PPI network of 79 Mtb TA systems. Our computational analysis revealed significantly enriched gene ontology terms for pathways and molecular functions of Mtb TA systems and topological properties of PPI network. The major contribution is the identification of hub proteins of PPI network that can be explored as promising drug targets and for vaccine development. In addition, homology models of hub proteins VapC9 and VapC10 provide insights to its molecular functions.

## Figures and Tables

**Table 1:** Topological parameters of PPI network determined by STRING v10.5 and Network analyzer plugin of cytoscape 3.5.0.

SOURCE	NETWORK STATISTICS	
STRING	Number of nodes	167
	Number of edges:	234
	Average node degree:	2.8
	Avg. local clustering coefficient	0.628
	Expected number of edges:	41
	PPI enrichment p-value	0
NETWORK ANALYZER	Number of nodes (excluding isolated nodes)	157
	Clustering coefficient	0.137
	Connected components	25
	Network diameter	5
	Network radius	1
	Shortest paths	470
	Characteristic path lengths	1.787
	Avg. number of neighbors	2.981
	Network density	0
	Isolated nodes	0
	Number of self loops	0
	Multi edge node pairs	0

**Table 2:** GO biological pathway enrichment analysis of PPI network for 79 MTb TA systems.

Pathway ID	Pathway description	Count in gene set	False discovery rate
GO:0040008	regulation of growth	60	1.46E-77
GO:0090305	nucleic acid phosphodiester bond hydrolysis	61	1.08E-61
GO:0090501	RNA phosphodiester bond hydrolysis	49	3.73E-60
GO:0045926	negative regulation of growth	34	2.65E-45
GO:0045927	positive regulation of growth	31	2.75E-38
GO:0090304	nucleic acid metabolic process	67	2.59E-35
GO:0050789	regulation of biological process	64	1.60E-34
GO:0016070	RNA metabolic process	57	3.61E-33
GO:0048519	negative regulation of biological process	39	5.55E-31
GO:0048518	positive regulation of biological process	33	5.89E-28
GO:2000112	regulation of cellular macromolecule biosynthetic process	29	5.82E-14
GO:0010468	regulation of gene expression	29	2.04E-13
GO:0051171	regulation of nitrogen compound metabolic process	29	2.39E-13
GO:0080090	regulation of primary metabolic process	29	1.22E-12
GO:0031323	regulation of cellular metabolic process	29	1.69E-12
GO:0019222	regulation of metabolic process	30	1.74E-12
GO:0006355	regulation of transcription, DNA-templated	23	6.73E-10
GO:0051252	regulation of RNA metabolic process	22	5.75E-09
GO:0017148	negative regulation of translation	6	9.06E-08
GO:0006417	regulation of translation	9	1.20E-07
GO:0051172	negative regulation of nitrogen compound metabolic process	10	7.44E-07
GO:2000113	negative regulation of cellular macromolecule biosynthetic process	9	1.42E-06
GO:0010605	negative regulation of macromolecule metabolic process	10	3.98E-06
GO:0010629	negative regulation of gene expression	9	6.92E-06
GO:0031324	negative regulation of cellular metabolic process	10	9.52E-06
GO:0009892	negative regulation of metabolic process	11	1.22E-05
GO:0009987	cellular process	68	2.21E-05
GO:0006401	RNA catabolic process	6	2.36E-05
GO:0090502	RNA phosphodiester bond hydrolysis, endonucleolytic	6	0.000136
GO:0008150	biological_process	74	0.000313
GO:0006402	mRNA catabolic process	3	0.00466
GO:0016075	rRNA catabolic process	3	0.00466
GO:0045727	positive regulation of translation	3	0.00466
GO:0051253	negative regulation of RNA metabolic process	5	0.0107

**Table 3:** GO molecular function enrichment of PPI network for 79 MTb TA systems revealed over-representation of 11 GO ontology terms.

Pathway ID	Pathway description	Count in gene set	False discovery rate
GO:0004518	nuclease activity	61	6.27E-62
GO:0004540	ribonuclease activity	49	7.72E-61
GO:0000287	magnesium ion binding	33	4.71E-17
GO:0046872	metal ion binding	48	7.36E-12
GO:0005488	Binding	74	1.39E-11
GO:0004519	endonuclease activity	15	7.51E-10
GO:0003677	DNA binding	24	3.91E-06
GO:0097351	toxin-antitoxin pair type II binding	5	5.88E-06
GO:0004521	endoribonuclease activity	6	0.000254
GO:0003674	molecular_function	72	0.00188
GO:0003676	nucleic acid binding	25	0.00196

**Table 4:** List of top 15% hub proteins identified in PPI network of Mtb TA systems by consensus of betweenness and radiality statistical measures.

Name of protein	Rv number	Rank by		Score by	
Betweeness	Radiality	Betweeness	Radiality
higA1	Rv1956	1	2	2871.367	4.362833
vapB45	Rv2018	2	1	2762.824	4.446609
vapB14	Rv1952	3	7	2189.49	4.201723
Rv2762c	Rv2762c	4	15	2100.5	4.001948
vapC1	Rv0065	5	3	1528.757	4.337055
vapC9	Rv0960	6	4	1235.921	4.311278
vapB11	Rv1560	7	9	1181.667	4.092169
vapC10	Rv1397c	8	8	1096.531	4.175946
relB	Rv1247c	9	6	1006.576	4.233945
vapC19	Rv2548	11	19	800	3.88595
vapC5	Rv0627	12	11	769.2976	4.047059
vapC11	Rv1561	15	13	678	4.014837
higA3	Rv3183	17	14	478.7214	4.008393
vapC26	Rv0582	18	5	452.619	4.253278
vapC3	Rv0549c	19	12	407.0119	4.03417
vapC39	Rv2530c	20	19	373.3333	3.88595
vapC38	Rv2494	20	19	373.3333	3.88595
relF	Rv2865	22	22	347.5	3.873061
vapC4	Rv0595c	25	16	272.7	3.956838

**Table 5:** Physico-chemical properties of VapC9 and VapC10.

Physicochemical properties	VapC9	VapC10
Theoretical pI	13858.96	14952.43
Molecular weight	8.84	10.95
Extinction coefficient	15595	19480
Instability index	32.85	47.8
Aliphatic index	116.93	102.71
Grand average of hydropathicity (GRAVY)	0.15	-0.008

**Table 6:** Model evaluation scores of VapC9 and VapC10.

Server	Parameter	VapC9	VapC10
PROCHECK	Most favored regions (%)	93.00%	84.50%
	Additionally allowed regions (%)	6.10%	12.90%
	Generously allowed regions (%)	0.90%	2.60%
	Disallowed regions (%)	0.00%	0.00%
	Overall G-factor (%)	-0.32	-0.27
SWISS-MODEL	Z-score	-2.172	-1.478
	Q-Mean score	0.543	0.609
	D-fire energy	-152.76	-155.58
ERRAT	Overall quality (%)	97.30%	93.50%
ProSA-web	Z score	-4.07	-3.64
ProQ	LG score	5.248	3.15
	MaxSub	0.232	0.046
MOLPROBITY	Cβ deviations > 0.25A˚ (%)	0.00%	0.00%
	Residues with bad bonds (%)	0.00%	0.00%
	Residues with bad angles (%)	0.89%	0.75%
ResProx	Predicted Resolution (Å)	1.375	1.964

## Abbreviations

TA: Toxin-antitoxin
Mtb: Mycobacterium tuberculosis
GO: Gene ontology
PPI: Protein-protein interaction
